# Risk factors for *Coxiella burnetii* antibodies in bulk tank milk from Danish dairy herds

**DOI:** 10.1186/1751-0147-55-80

**Published:** 2013-11-14

**Authors:** Jens Frederik Agger, Suman Paul, Anna-Bodil Christoffersen, Jørgen Steen Agerholm

**Affiliations:** 1 Department of Large Animal Sciences, Faculty of Health and Medical Sciences, University of Copenhagen, Groennegaardsvej 8, DK-1870 Frederiksberg C, Denmark; 2 National Veterinary Institute, Technical University of Denmark, Bülowsvej 27, DK-1870 Frederiksberg C, Denmark

**Keywords:** *Coxiella burnetii*, Cattle, Risk factors, Biosecurity

## Abstract

The aim was to identify risk factors associated with *Coxiella burnetii* antibody positivity in bulk tank milk (BTM) samples from 100 randomly selected Danish dairy cattle herds. Antibody levels were measured by an enzyme-linked immuno-sorbent assay. Before testing the herds, the farm managers were interviewed about hired labour, biosecurity, housing and herd health during the 12 months prior to the study. Variables considered important for *C. burnetii* antibody positivity in multivariable logistic regression analysis included the sharing of machines between farms (OR = 3.6), human contacts (OR = 4.2), artificial insemination by other people than artificial insemination technicians (OR = 7.7), routine herd health contract with the veterinarian (OR = 4.3) and hygiene precautions taken by veterinarians (OR = 5). In addition, herd size, hired labour, trading of cattle between farms, quarantine and use of calving and disease pens also showed significant association in univariable analysis. This study demonstrates that strict biosecurity is important for the prevention of infections with *C. burnetii*.

## Findings

Control of infectious diseases in livestock is to a great extent based on prevention of introducing infectious microorganisms into susceptible populations. Therefore, identification of risk factors plays a key role in the management of biosecurity at farm level and there are increasing demands on development of biosecurity plans in livestock production in the European Union. Herd risk factors for Q fever, a zoonotic infection caused by the bacterium *Coxiella burnetii*, have not been well studied. Although *C. burnetii* has been found almost worldwide and despite rather high prevalences in many cattle populations [1], there is still need for knowledge on how to protect uninfected cattle herds from becoming infected. Thus the aim of this study was to identify risk factors for Danish dairy cattle herds having *C. burnetii* antibodies in bulk tank milk (BTM).

A cross sectional design was used to study 100 randomly selected dairy herds among the 4785 milk producing Danish dairy herds mandatorily listed in the Danish Cattle Database [2]. Farmers of the selected herds were interviewed by telephone during 20–30 minutes using a standardized questionnaire with closed and semi-open-ended questions [Additional file 1]. The questions concerned the use of hired labour, the housing system, general health of the herd, and farm management routines generally known to be of importance for herd biosecurity. A BTM sample from each herd was examined for *C. burnetii* antibodies using the commercial CHEKIT Q fever Antibody ELISA test kit (IDEXX, Liebefeld-Bern, Switzerland). The test was based on *C. burnetii* inactivated phase 1 and 2 antigens and the results were expressed as sample-to-positive values and estimated as S/P = [(OD _sample_ – OD _negative control_) / (OD _positive control_ – OD _negative control_) × 100]. According to the manufacturer, S/P ≥ 40%, S/P < 30% and results in the interval 30% ≤ S/P < 40% were considered as positive, negative and intermediate respectively. However, in our risk factor analysis in logistic regression we dichotomized the test results as positive for samples with S/P ≥ 40% and as negative for samples with S/P < 40%, as recommended by the manufacturer. The prevalence of seropositive herds was 59%, as previously reported [2]. Fisher’s exact test was applied to test relationships between *C. burnetii* antibody status and all ordinal and dichotomized variables. To account for possible nonlinear relations, values of all continuous variables were categorized into biological meaningful classes when appropriate before further analysis. Variable associations with *P* ≤ 0.25 in univariable analyses were included in the following multivariable logistic regression. Backward elimination of nonsignificant variables (*P* > 0.05) was used to select the final model, and the values of Hosmer-Lemeshow goodness of fit test were used to validate the models. Correlations among the exposure variables were checked by Spearman’s correlation test to avoid collinearity. There was no significant (P ≤ 0.05) correlation and no variables were removed due to this.

Eighteen out of 49 variables that had *P* ≤ 0.25 in univariable analysis were included in the multivariable analysis. The final logistic regression model (Table 1) showed that the risk of a seropositive BTM sample increased if the herd shared equipment (machines) with other farms, if cattle were in contact with visitors, if artificial insemination (AI) was done by other people than AI technicians, if the herd had a routine herd health contract with the veterinarian, and if hygiene precautions (changing boots and/or clothes, etc.) were not taken by the veterinarian before entering the herd. The Hosmer-Lemeshow value for goodness-of-fit of the final model was considered acceptable (*P* = 0.86) [3].

**Table 1:** **Multivariable logistic regression model for risk factors jointly associated with bulk tank milk antibody positivity to** ***Coxiella burnetii*** **in 100 randomly selected Danish dairy cattle herds collected in February 2008**

**Variables**	**Respondents**		**Odd Ratio (95% CI)**	P-value*
	**Total**	**No. (%) positive**		
Sharing equipment (machines) with other herds				0.04
*Yes*	23	17 (73.91)	3.62 (1.03 - 12.76)	
*No*	77	42 (54.55)	1	
Animals’ contact with human visitors				0.01
*Yes*	70	47 (79.66)	4.17 (1.41 - 12.5)	
*No*	30	12 (20.34)	1	
Insemination by other than artificial insemination (AI) technician				0.01
*Yes*	13	12 (92.31)	7.69 (2.08 - 16.95)	
*No*	87	47 (54.02)	1	
Routine herd health contract with veterinarian				0.01
*Yes*	56	40 (71.43)	4.32 (1.51 - 12.36)	
*No*	44	19 (43.18)	1	
Hygienic precautions by veterinarian before entering the herd				0.004
*No*	61	40 (65.57)	5.00 (1.66 - 15.12)	
*Yes*	39	19 (48.72)	1	

*P-value for the significant addition of the variable given the other variables in the model.

The final multivariable model (Table 1) primarily contains factors associated with introduction of infection into a herd. The factors: animal contact with human visitors from outside the farm, AI insemination by other people than AI technician, and herd health contract for routine health evaluation of the herd by the veterinarian were associated with increased antibody positivity (IAP). The most likely explanation is probably that such persons act as mechanical vectors carrying *C. burnetii* bacteria into the herd as stated in a review by Woldehiwet [4]. We found that AI done by other people than AI technicians increased the risk of BTM antibody positivity. Danish farmers who want to perform AI on their own cattle need authorization based on a course offered by the AI associations and semen is provided by AI associations. So the finding is difficult to explain; also because consequent reduced access of AI technicians to the herd should probably have lowered the risk. Farms with a routine herd health contract with a veterinarian had higher OR of being antibody positive than farms without such a contract thus suggesting that the veterinarian might bring the bacterium into the farm. Hygiene precautions taken by veterinarian, i.e. changing boots and/or clothes were found significantly to reduce the risk of IAP. In a multilevel analysis of data from the same study, but with cow as the analytical unit, we also found that the hygienic precautions reduced the risk of antibody positivity [5]. The similarity in results between cow and herd level analysis corresponds with our estimation of correlation between BTM antibody level and the within herd seroprevalence (R^2^ = 0.36; P < 0.001) in a previous study [6] of a subsample of 12 of the same herds as in the present study. However, *Taurel et al*. [7] only found a moderate correlation (R^2^ = 0.15) between BTM antibody level and within herd seroprevalence. In the present study we also observed that sharing farm equipment (machines) with other farms was significantly associated with IAP. Although our study indicates the importance of hygienic precautions in relation to personnel and equipment, Taurel *et al*. [8] did not find such associations in French dairy cattle.

Herd size, stable type, number of workers, use of calving and disease pens, purchasing animals, and quarantine practice were also found to have positive associations with IAP in univariable analysis (results not shown). Other studies have also shown relationship between IAP and increasing herd size [5,9], and between IAP and loose housing system [5,10] although a single study did not find relationship between IAP and housing system [11]. The hypothesis behind IAP and loose housing systems is that random movements of animals increase the probability of contact between infected and non-infected and increased contact with a contaminated environment and hence increased risk of transmission.

Selection bias in this study was minimized by random sampling and selection bias due to non-participation was considered negligible. Misclassification bias may result from unknown sensitivity and specificity of the ELISA used. However, the ELISA test for *C. burnetii* antibodies based on individual animal samples shows high sensitivity (Se = 0.86) and specificity (Sp = 0.99) [12].

In this study the following risk factors were found to be associated with increased risk of BTM antibody positivity: Herd size > 100 cows, increased number of people managing the animals; housing systems with cubicle house and deep bed house compared to tie stall house, use of calving and disease pens, purchase of animals, lack of quarantine of purchased animals, contact with farm personnel and visitors, routine herd health contract with the veterinarian, lack of basic hygienic precautions taken by veterinarian and sharing of machines. Proper management by farmers may help prevent the introduction of *C. burnetii* into a herd.

## Abbreviations

AI: Artificial insemination; BTM: Bulk tank milk; ELISA: Enzyme-linked immuno-sorbent assay; IAP: Increased antibody positivity.

## Competing interests

JSA is editor-in-chief of Acta Veterinaria Scandinavia, but has not in any way been involved in or interacted with the review process or editorial decision making. The authors declare they have no competing interests.

## Authors’ contribution

JFA, ABC and JSA designed the study and developed the questionnaire. JFA selected the herds and conducted the interviews. ABC was responsible for sampling of milk and conducted the laboratory analysis. SP and JFA conducted the analysis and drafted the manuscript. All authors commented and approved the final manuscript.

## Supplementary Material

Additional file 1 Questionaire used for telephone interview. Click here for file
