# Supplementary material for: Risk factors for Coxiella burnetii antibodies in bulk tank milk from Danish dairy herds
Source: Acta Vet Scand. 2013 Nov 14;55(1):80. doi: 10.1186/1751-0147-55-80 (PMC4176746; doi:10.1186/1751-0147-55-80)
Supplement: Additional file 1 — Questionaire used for telephone interview. [file 1751-0147-55-80-S1.doc]

**Questionnaire used in telephone interviews of dairy herd owner/manager for collecting herd level information.**

**Employee:**

**Responsible:**

Jens Frederik Agger, Associate Professor, Department of Large Animal Sciences, Section for Population Biology (Epidemiology), LIFE, University of Copenhagen. Gronnegaardsvej 8, DK-1870 Frederiksberg C.

**Student Employee:**

Lærke Boye Thomsen, student of Veterinary Medicine.

*Remember that courtesy is a matter of course.* *We would indeed like to come back. ☺*

**Name of interviewer:**

Name: ___________________________________________________

| **General information** | | |
| --- | --- | --- |
| Date of interview | : | ____ /____ / 2008 |
| Project number (1-150) | : | _________________________________ |
| CHR number | : | _________________________________ |
| Telephone number | : | _________________________________ |
| Farmer’s name / address | : | _________________________________ |

**Introduction:**

Good afternoon / evening, my name is xxx.

I'm calling from the Faculty of Life Sciences, University of Copenhagen, where we conduct a study of Q fever in Danish dairy cattle herds. A few days ago the Dairy Board sent you a letter with an invitation to participate in this study.

| 1. Have you received this letter? (If NO, we need to explain why we called. Then they probably say YES). | : | YES | NO |
| --- | --- | --- | --- |
|  | | | |
|  | | | |
| 2. Are you willing to participate in this study? | : | YES | NO |
| **If NO**  3. Are there any special reasons why you do not want to participate?  __________________________________________________________________________________________________________________________________________________________________________________________  Sorry for the inconvenience and have a good day / evening. | | | |

**If YES**

**Thank you. You will be attended soon.**

We will shortly send you a plastic tube to take a milk sample from your bulk milk tank. The sample is to be send to the National Veterinary Institute, Technical University of Denmark.

In the letter, there will be instructions on how the sample must be taken and send to the laboratory. We will enclose a stamped addressed envelope.

I would like to ask you some questions about your farm. It will take 20-30 minutes.

Questions about the herd

| **About number of employees**: | | | | | | | | | | |
| --- | --- | --- | --- | --- | --- | --- | --- | --- | --- | --- |
| 4. How many people help in milking, feeding and caring for your animals (cows, heifers, old calves and young calves)? | | | | | | | | : | Number: | |
|  | | | | | | | | | | |
| **About stables and health of animals** | | | | | | | | | | |
| 5. What type of housing do you use for the cows? | | | | | | | | | | |
| Tie stall house | | | Cubicle house | | | Deep bed house | | | | |
| Further elaboration here:  _____________________________________________________________ | | | | | | | | | | |
| 6. Where do the cows calve in the herd? | | | | | | | | | | |
| Calving pen | | | | Tie stall | | | Cubicle or deep bed | | | |
|  | | | | | | | | | | |
| 7. Do you have a disease pen for sick cows? | | | | | | | | | | |
| YES | | YES, but it also serves as a calving pen | | | | | | | | NO |
|  | | | | | | | | | | |
| 8. Have there been changes in the pattern of disease in cows during the last 6 months compared with earlier times? | | | | | | | | | | |
|  | **Syndrom** | | | | **YES** | | | **NO** | | |
|  | Abortions | | | |  | | |  | | |
|  | Stillborn calves | | | |  | | |  | | |
|  | Weak-born calves | | | |  | | |  | | |
|  | Retained placenta | | | |  | | |  | | |
|  | Endometritis | | | |  | | |  | | |
|  | Reproductive problems | | | |  | | |  | | |
|  | Other | | | |  | | |  | | |
| 9. Have there been changes in the pattern of disease in old calves/heifers the last 6 months compared with earlier times? | | | | | | | | | | |
| YES | | | | | NO | | | | | |
| **If YES**, please specify: ______________________________________________  ______________________________________________________________ | | | | | | | | | | |
|  | | | | | | | | | | |
| 10. Have there been changes in the pattern of disease in young calves the last 6 months compared with earlier times? | | | | | | | | | | |
| YES | | | | | NO | | | | | |
| **If YES,** please specify: ______________________________________________  ______________________________________________________________ | | | | | | | | | | |

| **Biosecurity procedures, including contact with other herds** | | | | | | |
| --- | --- | --- | --- | --- | --- | --- |
| 11. Have you purchased cattle to the herd during the last 12 months? | | | | | | |
| Number of purchased cows | | | Number of purchased heifers  and old calves | | | |
| Number of purchased young calves | | |  | | | |
| 12. How many suppliers have delivered the purchased cattle? | | | | | | |
| 13. How many suppliers have a herd health advisory contract with the veterinarian? | | | | | | |
| Number | | | Don’t know | | | |
| 14. Do you buy cattle from the assembly sites (e.g. livestock auctions)? | | | | | | |
| YES | | | NO | | | |
| 15. Do you bring your animals to shows or other exhibitions? | | | | | | |
| YES | | | NO | | | |
| 16. Do you buy fodder from other herds? | | | | | | |
| YES | | | NO | | | |
| 17. Do you buy straw or bedding material from other herds? | | | | | | |
| YES | | | NO | | | |
| 18. How often you visit other herds or stables? | | | | | | |
| NEVER | RARELY | | | | | OFTEN |
| 19. How often do other farmers visit your herd or stables? | | | | | | |
| NEVER | RARELY | | | | | OFTEN |
| 20. Do you share machines with other herds? | | | | | | |
| YES, cattle trucks | | | | YES, field machines | | |
| YES, Other Specify:  ___________________________________ | | | | | | |
| NO | | | |  | | |
| 21. Who inseminates the cows in your herd? | | | | | | |
| Artificial insemination (AI) technician | | | | Veterinarian | | |
| By own bull | | | | Other | | |
| **If other,** please specify:___________________________________________ | | | | | | |
| 22. Who is (are) the herd veterinarian(s)? | | | | | | |
| Name : ________________________________________________________  ______________________________________________________________ | | | | | | |
| 23. Do you have a herd health agreement contract with your veterinarian? | | | | | | |
| YES | | | | NO | | |
| 24. What other people get in contact with your cattle? | | | | | | |
| Livestock consultant | | | | Truck driver | | |
| Other Specify:____________________________ | | | | | | |
| 25. Do you bring cattle on pasture during summer? | | | | | | |
| YES | | | | NO (if No, go to question 27) | | |
| 26. **If YES to question 24:**  Do your cattle come into physical contact with animals in neighboring fields (across a common fence)? | | | | | | |
| YES | | | | NO | | |
| Describe: _______________________________________________________  ______________________________________________________________ | | | | | | |
| 27. Do you bring cattle on common pasture with cattle from other herds? | | | | | | |
| YES | | | | NO | | |
| Describe: _______________________________________________________  ______________________________________________________________ | | | | | | |
| 28. Have you prohibited other people from entering your stable without your permission? | | | | | | |
| YES | | | | NO | | |
| 29. Do you require hygiene and biosecurity precautions before people enter your stable? | | | | | | |
| Yes | | | | NO | | |
| **If Yes,** please describe: _____________________________________________ | | | | | | |
| 30. Do you quarantine purchased supply cattle? | | | | | | |
| Yes | | NO | | | Don’t buy new animals | |
| Explain: _______________________________________________________  ______________________________________________________________ | | | | | | |
| 31. Which hygienic measures does the herd veterinarian take before entering the stable? | | | | | | |
| Nothing | | | Change clothes | | | |
| Wash/change boots | | |  | | | |
| 32. What hygienic measures does the herd veterinarian take at the end of the visit? | | | | | | |
| Nothing | | | Change clothes | | | |
| Wash/change boots | | |  | | | |
| 33. What hygienic measures does the AI technician take before entering the stable? | | | | | | |
| Nothing | | | Change clothes | | | |
| Wash/change boots | | |  | | | |
| 34. What hygienic measures does the AI technician take at the end of the visit? | | | | | | |
| Nothing | | | Change clothes | | | |
| Wash/change boots | | |  | | | |
| 35. What hygienic measures does the livestock consultant take before entering the stable? | | | | | | |
| Nothing | | | | Change clothes | | |
| Wash/change boots | | | | Does not enter the stable | | |
| 36. What hygienic measures does the livestock consultant take at the end of the visit? | | | | | | |
| Nothing | | | | Change clothes | | |
| Wash/change boots | | | | Does not enter the stable | | |
| 37. What hygienic measures does the truck driver take before entering the stable? | | | | | | |
| Nothing | | | | Change clothes | | |
| Wash/change boots | | | | Does not enter the stable | | |
| 38. What hygienic measure does the animal consultant take at the end of the visit? | | | | | | |
| Nothing | | | | Change clothes | | |
| Wash/change boots | | | | Does not enter the stable | | |
| 39. What hygienic measure do OTHER people take before entering the stable?  Specify who is (are) the OTHER(S):_____________________________________ | | | | | | |
| Nothing | | | | Change clothes | | |
| Wash/change boots | | | | Do not enter the stable | | |
| 40. What hygienic measure do OTHER people take when finishing the visit? | | | | | | |
| Nothing | | | | Changes cloths | | |
| Washes/changes boots | | | |  | | |
| 41. Do you disinfectant foot ware before entering the stable? | | | | | | |
| Yes | | | | No | | |
| 42. Do you in other ways take action to protect the herd against infection? | | | | | | |
| YES | | | | NO | | |
| Explain: ________________________________________________________  ______________________________________________________________ | | | | | | |
